# Differences in respiratory consultations in primary care between underweight, normal-weight, and overweight children

**DOI:** 10.1038/s41533-019-0131-0

**Published:** 2019-05-03

**Authors:** Janneke van Leeuwen, Zoubeir El Jaouhari, Winifred D. Paulis, Patrick J. E. Bindels, Bart W. Koes, Marienke van Middelkoop

**Affiliations:** 1 Department of General Practice, Erasmus MC, University Medical Center, Rotterdam, The Netherlands; 2 Department of Physical Therapy Studies, Rotterdam University of Applied Sciences, Rotterdam, The Netherlands

**Keywords:** Asthma, Respiratory signs and symptoms

## Abstract

This prospective cohort study investigates whether the suggested association between weight status and respiratory complaints in open populations is also reflected in the frequency of consultations for respiratory complaints at the general practice. Children aged 2–18 years presenting at one of the participating general practices in the Netherlands could be included. Electronic medical files were used to extract data on consultations. Logistic regression analyses and negative binomial regression analyses were used to assess the associations between weight status and the presence, and frequency of respiratory consultations, respectively, during 2-year follow-up. Subgroup analyses were performed in children aged 2–6, 6–12, and 12–18 years old. Of the 617 children, 115 (18.6%) were underweight, 391 (63.4%) were normal-weight, and 111 (18%) were overweight. Respiratory consultations were not more prevalent in underweight children compared to normal-weight children (odds ratio (OR) 0.87, 95% confidence inteval (CI) 0.64–1.10), and in overweight children compared to normal-weight children (OR 1.33, 95% CI 0.99–1.77). Overweight children aged 12–18 years had more respiratory consultations (OR 2.14, 95% CI 1.14–4.01), more asthma-like consultations (OR 3.94, 95%CI 1.20–12.88), and more respiratory allergy-related consultations (OR 3.14, 95% CI 1.25–7.86) than normal-weight children. General practitioners should pay attention to weight loss as part of the treatment of respiratory complaints in overweight and obese children.

## Introduction

Pediatric underweight, overweight, and obesity are, among other diseases, associated with respiratory diseases and symptoms, such as asthma and allergic rhinitis.^1–5^ Previous studies have shown a u-shaped association between weight status and prevalence of asthma.^3,5^ Several underlying mechanisms have been suggested for the higher prevalence of asthma in obese children.^6,7^ One of these include that high body weight may exacerbate airway inflammation, which may also contribute to the development of asthma.^8^ Symptoms of asthma in overweight and obese children are also partly due to the excess weight itself, and its accompanying fat deposition in the upper body, abdomen, and upper airways.^9^ Besides asthma, obesity is also linked to atopy, like allergic rhinitis; however, the evidence for this association is contradicting.^8,10,11^

In 2016, worldwide 14% of children under the age of 5 years was underweight, and just over 18% of children aged 5–19 years was overweight or obese.^12,13^ In 2016, in the Netherlands, 7.4% of children aged 4–12 years was underweight,^14^ 13.6% of children aged 4–17 years was overweight, and 2.7% was obese.^15^ Although the prevalence of underweight has slowly declined in the past decennia, the prevalence of overweight and obesity has steadily increased over the past years both worldwide and in the Netherlands.^13,15,16^

Asthma is among the top two diseases in the Netherlands for which children consult the general practitioner (GP) the most.^17^ In the Netherlands, the GP is responsible for primary care and therefore the first doctor to assess a symptom or health complaint. Up to now, studies that stated that weight status is associated with different kind of respiratory diseases and symptoms were all conducted in open-based or school-based populations and used questionnaires to gather data on symptoms and diseases.^1–5^ Therefore, the question arises whether the suggested association between weight status and respiratory complaints in an open population is reflected in the frequency of consultations for respiratory complaints at the GP by underweight, normal-weight, and overweight children.

This study investigated the association between weight status and the number of respiratory consultations in general, and specific respiratory consultations at the GP, which include asthma-like consultations, respiratory inflammatory consultations, and respiratory allergy-related consultations. Since the prevalence of asthma and other respiratory symptoms in children varies between different ages, the current study also investigated the beforementioned associations in different age categories.^18^

## Results

### General characteristics

**Fig. 1:**
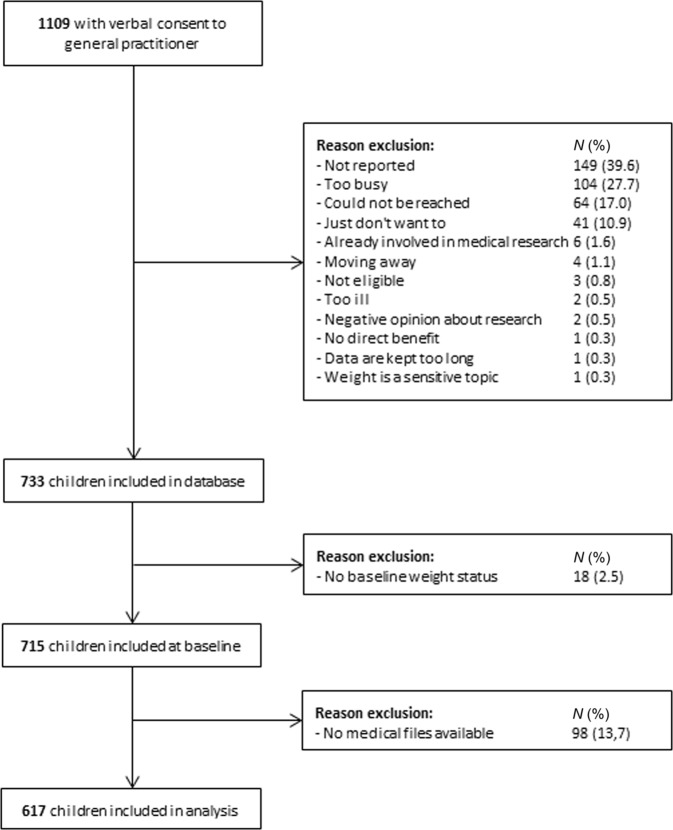
Flowchart of inclusion

Of the 1109 children who initially gave verbal consent to participate in the study, 733 gave written consent and were included in the database (Fig. 1). Children with missing baseline weight status (*n* = 18) and/or children who did not give permission to review their medical files (*n* = 98) were excluded from the analysis; therefore; 617 children were included in the analyses. The excluded children were significantly older (mean 9.45 (SD 4.4) vs. 7.96 (3.9), *p* = 0.001)) and had a higher BMI *z*-score at baseline (0.49 (1.3) vs. 0.06 (1.3), *p* = 0.003) compared to included children.

At baseline, 115 (18.6%) children were underweight, 391 (63.4%) were of normal-weight, and 111 (18.0%) were overweight (Table 1). Underweight children were younger (6.77(3.8) vs. 8.05(4.0), *p* = 0.017) and more often breastfed (85.4% vs. 68.6%, *p* = 0.025) than normal-weight children. Parents of overweight children were less often from Dutch descent compared to parents of normal-weight children (79.8% vs. 86.3%, *p* = 0.006).

**Table 1:** Baseline characteristics

	Study population (*n* = 617)	Underweight (*n* = 115; 18.6%)	Normal-weight (*n* = 391; 63.4%)	Overweight (*n* = 111; 18.0%)
Gender, female (*n*; %)	321 (52.0)	61 (53.0)	199 (50.9)	61 (55.0)
Age (mean; SD)	7.96 (3.9)^a^	6.77 (3.8)^b^	8.05 (4.0)	8.87 (3.6)
Ethnicity (*n*; %)				
Both parents born in the Netherlands	458 (84.8)	90 (84.9)	289 (86.3)	79 (79.8)^c^
At least one parent not born in the Netherlands	82 (15.2)	16 (15.1)	46 (13.7)	20 (20.2)
SES (*n*; %)				
Low (<2000 euros)	110 (21.4)	19 (19.4)	69 (21.4)	22 (23.9)
Middle/high (≥2000 euros)	403 (78.6)	79 (80.6)	254 (78.6)	70 (76.1)
Marital status (*n*; %)				
Parents together	463 (84.0)	90 (84.9)	289 (84.3)	84 (82.4)
Parents not together	88 (16.0)	16 (15.1)	54 (15.7)	18 (17.6)
Education parents (*n*; %)				
Up to lower level secondary education	91 (16.5)	19 (17.9)	56 (16.3)	16 (15.5)
Higher level secondary education	222 (40.1)	35 (33.0)	141 (41.0)	46 (44.7)
At least a bachelor diploma	240 (43.4)	52 (49.1)	147 (42.7)	41 (39.8)
Breastfeeding (*n*; %)				
Breastfed	352 (71.4)	82 (85.4)^b^	214 (68.6)	56 (65.9)
Not breastfed	141 (28.6)	14 (14.6)^b^	98 (31.4)	29 (34.1)
Birth weight (mean; SD)	3421 (632)	3333 (550)	3413 (625)	3548 (725)
BMI *z*-score (mean; SD)	0.06 (1.3)^a^	−1.79 (0.9)^b^	0.06 (0.7)	1.98 (0.7) ^c^

*SES* socioeconomic status, *BMI* body mass index

^a^Significant difference between analysis group and group excluded from analysis, *p* < 0.05

^b^Significant difference between normal-weight and underweight, *p* < 0.05

^c^Significant difference between normal-weight and overweight, *p* < 0.05

### Overall prevalence of consultations

Children had a mean of 6.9 (SD 5.6) consultations of any type during the 2-year follow-up. No significant differences were seen in the number of consultations between underweight (6.8 (5.1)) and normal-weight (6.7 (5.7)) children (odds ratio (OR) 1.04, 95% confidence interval (CI) 0.83–1.31), and between overweight (7.3 (5.7)) and normal-weight children (OR 1.10, 95% CI 0.88–1.39).

### Presence of respiratory consultations during 2-year follow-up

During the 2-year follow-up, 570 (92.4%) children consulted the GP at least once for any type of complaint and 279 (45.2%) children consulted the GP at least once for a respiratory complaint (Table 2). For asthma-like complaints, respiratory inflammatory complaints, and respiratory allergy-related complaints, 47 (7.6%), 168 (27.2%), and 81 (13.1%) children, respectively, consulted the GP at least once during the 2-year follow-up. There were no significant differences in the number of children with respiratory consultations between underweight and normal-weight children (OR 1.06, 95% CI 0.68–1.63), and overweight and normal-weight children (OR 1.54, 95% CI 0.99–2.39). No significant differences between weight status groups were found for the number of children consulting the GP with asthma-like complaints, respiratory inflammatory complaints, and respiratory allergy-related complaints.

**Table 2:** Number of children with at least one respiratory consultation during the 2-year follow-up, and the number of respiratory consultations per child during 2-year follow-up

	Study population (*n* = 617)	Normal-weight (*n* = 391)	Underweight (*n* = 115)	Adjusted OR (95% CI) ^a^	Overweight (*n* = 111)	Adjusted OR (95% CI)^b^
Number of children with respiratory consultations						
Any respiratory consultation, *n* (%)	279 (45.2)	168 (43.0)	53 (46.1)	1.06 (0.68–1.63)	58 (52.3)	1.54 (0.99–2.39)
Asthma-like consultations, *n* (%)	47 (7.6)	24 (6.1)	12 (10.4)	2.01 (0.94–4.30)	11 (9.9)	1.59 (0.74–3.42)
Respiratory inflammatory consultations, *n* (%)	168 (27.2)	108 (27.6)	32 (27.8)	0.87 (0.53–1.42)	28 (25.2)	0.98 (0.59–1.63)
Respiratory allergy-related consultations, *n* (%)	81 (13.1)	46 (11.8)	15 (13.0)	1.30 (0.98–2.49)	20 (18.0)	1.53 (0.84–2.77)
Number of respiratory consultations per child						
Any respiratory consultation, mean (s.d.)	1.2 (2.0)	1.2 (2.0)	1.0 (1.6)	0.87 (0.64–1.1)	1.4 (2.2)	1.33 (0.99–1.77)
Asthma-like consultations, mean (s.d.)	0.2 (0.8)	0.2 (0.7)	0.2 (0.7)	1.51 (0.85–2.69)	0.3 (1.0)	1.59 (0.94–2.68)
Respiratory inflammatory consultations, mean (s.d.)	0.5 (1.1)	0.5 (1.1)	0.5 (1.1)	0.79 (0.54–1.16)	0.4 (0.9)	0.85 (0.57–1.27)
Respiratory–allergy-related consultations, mean (s.d.)	0.3 (0.9)	0.2 (0.8)	0.2 (0.7)	1.17 (0.70–1.97)	0.4 (1.1)	1.65 (1.06–2.57)*

*SES* socioeconomic status, *OR* odds ratio, *CI* confidence interval

*P < 0.05

^a^OR between normal-weight and underweight, adjusted for gender, age, ethnicity, SES, and breastfeeding

^b^OR between normal-weight and overweight, adjusted for gender, age, ethnicity, SES, and breastfeeding

### Number of respiratory consultations

Children had a mean of 1.2 (2.0) respiratory consultations during the 2-year follow-up (Table 2). No significant differences were seen in the number of respiratory consultations between underweight (1.0 (1.6)) and normal-weight (1.2 (2.0)) children (OR 0.87, 95% CI 0.64–1.10), and between overweight (1.4 (2.2)) and normal-weight children (OR 1.33, 95% CI 0.99–1.77). Though overweight children consulted the GP significantly more often for respiratory allergy-related consultations (0.4 (1.1)) than normal-weight (0.2 (0.8)) children (OR 1.65, 95% CI 1.06–2.57). No significant differences between the weight status groups were found for asthma-like consultations and respiratory inflammatory consultations.

### Respiratory consultations per age category

The association between weight status and number of respiratory consultations during 2-year follow-up was investigated in three different age categories (2–6, 6–12, 12–18 years) (Table 3). This analysis revealed that overweight children aged 12–18 years had significantly more respiratory consultations at the GP (1.87 (3.06) vs. 0.93 (1.54)) than normal-weight children aged 12–18 years (OR 2.14, 95% CI 1.14–4.01).

**Table 3:** Number of respiratory consultation per child during 2-year follow-up per age category

	Age category	Normal-weight		Underweight		OR (95% CI)^a^	Overweight		OR (95% CI)^b^
		*N* (%)	Mean (SD)	*N* (%)	Mean (SD)		*N* (%)	Mean (SD)	
Any respiratory consultation	2–6 years	121 (30.9)	1.88 (2.73)	55 (47.8)	1.31 (1.85)	0.74 (0.48–1.16)	18 (16.2)	2.06 (2.34)	1.15 (0.61–2.14)
	6–12 years	190 (48.6)	0.81 (1.87)	45 (39.1)	0.69 (1.08)	0.87 (0.52–1.45)	70 (63.1)	1.09 (1.76)	1.30 (0.86–1.97)
	12–18 years	80 (20.5)	0.93 (1.54)	15 (13.1)	1.00 (1.96)	1.07 (0.45–2.54)	23 (20.7)	1.87 (3.06)	2.14 (1.14–4.01)*
Asthma-like consultations	2–6 years	121 (30.9)	0.12 (0.67)	55 (47.8)	0.20 (0.87)	1.91 (0.71–5.11)	18 (16.2)	0.06 (0.24)	0.57 (0.07–4.77)
	6–12 years	190 (48.6)	0.15 (0.64)	45 (39.1)	0.13 (0.40)	0.77 (0.29–2.10)	70 (63.1)	0.24 (0.95)	1.34 (0.62–3.55)
	12–18 years	80 (20.5)	0.20 (1.00)	15 (13.1)	0.27 (0.80)	3.17 (0.53–19.12)	23 (20.7)	0.48 (1.50)	3.94 (1.20–12.88)*
Respiratory inflammatory consultations	2–6 years	121 (30.9)	0.99 (1.52)	55 (47.8)	0.65 (1.31)	0.71 (0.42–1.19)	18 (16.2)	0.94 (1.76)	0.97 (0.46–2.01)
	6–12 years	190 (48.6)	0.31 (0.71)	45 (39.1)	0.33 (0.77)	1.08 (0.56–2.11)	70 (63.1)	0.33 (0.72)	1.13 (0.64–2.01)
	12–18 years	80 (20.5)	0.31 (0.80)	15 (13.1)	0.20 (0.56)	0.50 (0.12–2.09)	23 (20.7)	0.22 (0.52)	0.74 (0.24–2.32)
Respiratory allergy-related consultations	2–6 years	121 (30.9)	0.16 (0.70)	55 (47.8)	0.20 (0.87)	1.33 (0.53–3.34)	18 (16.2)	0.17 (0.71)	1.22 (0.31–4.83)
	6–12 years	190 (48.6)	0.23 (0.73)	45 (39.1)	0.20 (0.50)	0.79 (0.34–1.82)	70 (63.1)	0.34 (0.99)	1.21 (0.63–2.30)
	12–18 years	80 (20.5)	0.31 (1.05)	15 (13.1)	0.33 (0.82)	2.14 (0.47–9.77)	23 (20.7)	0.78 (1.68)	3.14 (1.25–7.86)*

*SES* socioeconomic status, *OR* odds ratio, *CI* confidence interval

*p < 0.05

^a^OR between normal-weight and underweight, adjusted for gender, ethnicity, SES and breastfeeding

^b^ OR between normal-weight and overweight, adjusted for gender, ethnicity, SES, and breastfeeding

Overweight children aged 12–18 years also had more asthma-related consultations (0.48 (1.50) vs. 0.20 (1.00)) (OR 3.94, 95% CI 1.20–12.88), and more respiratory allergy-related consultations (0.78 (1.68) vs. 0.31 (1.05)) (OR 3.14, 95% CI 1.25–7.86) than normal-weight children aged 12–18 years.

No further significant differences were seen between weight status, specifically between underweight and normal-weight children, and the number of (further specified) respiratory consultations in the different age categories.

## Discussion

Overall, no significant differences were found in the number of children (aged 2–18 years) visiting the GP with at least one respiratory complaint between underweight and normal-weight children, and overweight and normal-weight children during 2 years of follow-up. Overweight children of all ages consulted the GP more often than normal-weight children (0.4 vs. 0.2) only for respiratory allergy-related consultations. However, overweight children aged 12–18 years had significantly more respiratory consultations in general (2.14 vs. 0.93), asthma-like consultations (3.94 vs. 0.20), and respiratory allergy-related consultations (3.14 vs. 0.31) than normal-weight children of this age.

We found that older overweight children had significantly more asthma-like consultations than their normal-weight peers. This is comparable with the results from a review, which supports the association between overweight and asthma as well.^2^ Moreover, two studies included in this review showed that with increasing age, the OR between overweight and asthma increased, which is similar to what we found.^19,20^ This may strengthen the suggestion that the relationship between obesity and asthma is dose-dependent, since older children who became overweight at an early age have been exposed to obesity for a longer period.^8^ They have also been exposed to metabolic dysregulation and mechanical factors such as excess truncal adiposity for a longer period, and therefore may have more asthma complaints for which they consult the GP.^6–8^

Another explanation for the fact that we only found an association between obesity and asthma in older children is that a recent review, including 21,130 children, suggested that the association between asthma and obesity may be inverse, meaning that asthma may lead to obesity.^21^ This association may partially be explained by lifestyle factors, that is, asthmatic children have lower levels of physical activity and less sleep than healthy children, both of which can lead to obesity.^22^ These processes may take a few years, which may explain that the association between obesity and asthma in our study is only found in older children.

The fact that that the association between obesity and asthma in our study was only found in older children may also be explained by the influence of hormonal factors on respiratory symptoms. It has been shown that obesity and early onset of puberty are independent risk factors for persistence of asthma after the onset of puberty in both boys and girls.^23^ Furthermore, early menarche at an age under 11.5 years predicts post-menarcheal incidence of asthma.^24^

In addition to the higher percentage of asthma-like symptoms found in overweight children aged 12–18 years, significantly more respiratory and more respiratory allergy related consultations were seen in overweight children aged 12–18 years compared to their normal-weight peers. This difference was, however, not present in children aged 2–6 and 6–12 years old. This phenomenon may again be explained by the suggested dose-dependent relationship between obesity and asthma.^8^ It could also be suggested that the low percentage of overweight children aged 2–6 years in the study population (only 9%) introduced a statistical power problem. However, when looking at the OR’s and the 95% CI of the associations between weight status and respiratory consultations in children aged 2–6 years old, they are not close to significance. Therefore, it does not seem that the fact that there were no differences in respiratory consultations between the young overweight and normal-weight children can be explained by lack of power.

It was notable that in our sample 18.6% of the children was underweight based on the age-specific and gender-specific cut-off scores from Cole et al.,^14^ while in 2016 in the Netherlands 7.4% of children aged 4–12 years was underweight. We therefore wanted to investigate whether this large proportion of underweight children had an impact on our results. To approach the underweight percentage of 7.4% in the Netherlands, we manually adjusted the cut-off so that only the 7.5% most underweight children in our sample were marked as underweight. When re-running the analyses with this stricter cut-off, still no significant differences between underweight and normal-weight children for respiratory consultations were found. Therefore, we believe that the results found in the underweight children are valid.

We earlier showed that overweight children consult their GP more often than normal-weight children.^25^ The current study found that this difference may partly be explained by the increased number of respiratory consultations seen in the older overweight children. However, the clinical relevance of this difference is questionable. Extrapolating the number of asthma-related consultations in the current study to the Dutch population, a GP in the Netherlands will have about four asthma-related consultations per year from 17 overweight children aged 12–18 years, compared to 11 consultations per year from 109 normal-weight children aged 12–18 years.^14^ On the other hand, in our cohort, 19% of all consultations were from children with overweight, while 35% of asthma-like consultations were from children with overweight. Thus, although the absolute number of normal-weight and overweight children consulting the GP for asthma complaints may not differ much, relatively, the percentage of respiratory consultations from children with overweight is much larger than the percentage of all type of consultations from children with overweight.

The asthma clinical guideline for GPs does not differentiate between the treatment of asthma in normal-weight or overweight children.^26^ There is some evidence that suggests that weight loss may lessen asthma symptom severity.^2,27^ Therefore, it could be suggested that implementation of weight loss treatment for overweight and obese children in the asthma clinical guideline may be beneficial for the treatment of asthma. However, more evidence on the effectiveness of weight loss is mandatory in order to implement these recommendations in clinical guidelines.

This study is the first to investigate the association between children’s weight status and frequency and type of respiratory consultations at the GP. Weight and height of the children were measured by GPs, rather than using self-reported measures, which increases the reliability of these measures. Medical files to extract data on the number and type of consultations at the GP were used, instead of using questionnaires, which is an important strength of this study, as this means we are not confronted with recall bias.

One limitation is that the study population was smaller than initially anticipated,^28^ which may have introduced a power problem, especially in the younger children. By instructing the GPs to invite every child who presented himself or herself at the GP during the inclusion period to participate in the study, we tried to minimize selection bias. However, when we compare our study population to the overall Dutch population, we found that our study population includes less families with an ethnic minority background (15.2% vs. 22.6%), and more families with a high level of education (43% vs. 32%). Therefore, our cohort may not be completely representative of all children in general practices. Furthermore, the excluded children in our study were significantly older and had a significantly higher BMI *z*-score than included children. Since we found in the current study that it is mostly the older overweight children that consult the GP more often for different types of respiratory consultations, the exclusion of these children could have led to an underestimation of the amount of respiratory consultations at the GP.

In conclusion, overweight children aged 2–18 years consulted the GP more often than normal-weight children for respiratory allergy-related consultations. Overweight children aged 12–18 years consulted the GP more often for respiratory symptoms, asthma-like symptoms, and respiratory allergy-related symptoms than their normal-weight peers. Since evidence suggests that weight loss may lessen asthma symptom severity, there may be a place for weight loss treatment for overweight children in the asthma clinical guideline.^2,27^ However, this effectiveness of weight loss on asthma symptoms should first be further investigated before these recommendations may be implemented.

## Methods

### Study design

This study was a prospective cohort study with a follow-up of 2 years; data from the DOERAK (Determinants of (sustained) Overweight and complaints; Epidemiological Research among Adolescents and Kids in general practice) study were used.^28^ The Institutional Review Board of the Erasmus University Medical Center has approved the DOERAK study (MEC-2010-092).

### Participant selection

GPs, and GP trainees in their last year of education (from now on both GP), invited all children who consulted the GP between December 2010 and April 2013 for any type of complaint to participate in the study. These children could be invited at 71 participating GP offices located in various socioeconomic regions in the South-West of the Netherlands. Children had to be aged 2–18 years and both children, depending on their age, and their parent(s) had to have at least a basic understanding of the Dutch language to be able to give informed consent and understand the questionnaires. Children who were disabled, had serious comorbidities affecting weight, or consulted the GP for an emergency were excluded.

Eligible children and their parents received verbal information from their GP during consultation. If they were interested to participate in the study, the child’s height and weight were measured by the GP. Hereafter, written information and an informed consent form was provided to the parents, and an informed assent form was provided to children aged 12 years and older. Within 2 weeks, the family was contacted by the research assistant to answer any remaining questions and to examine their interest to participate in the study. Once the signed informed consent forms (and if applicable, assent forms) were received, the child was officially included in the study. During the study period, children received usual care from their GP.

### Data collection

Height and weight were measured by the GP at baseline and an online questionnaire with questions about, among other things, sociodemographic information was sent to parents and, if at least 9 years old, to the child. Families without access to internet received paper copies of the questionnaires via post. If the questionnaires were not completed 1 week after the participant received the questionnaire, weekly reminders were sent to the child and/or parents.

Information regarding the frequencies and types of consultations of the children at the GP during the 2-year follow-up was registered in the electronic medical files at the GP office. In these medical files, reasons for consultation and the accompanying diagnoses were recorded by the GP using the International Classification of Primary Care (ICPC) (Supplementary Table 1).^29^ These ICPC codes, together with possible explanatory comments, were extracted for analysis from the electronic medical files.

### Measures

Age and gender were extracted from the GPs’ baseline questionnaires. Weight status was determined based on body mass index (BMI) *z*-scores, which were calculated using BMI (body weight in kilograms divided by height in meters squared) and age-specific and gender-specific cut-off points.^30,31^ Due to the small number of obese children in the cohort (*n* = 24), both obese and overweight children were merged into the overweight group, classified as BMI >85th percentile.

Ethnicity, parental education, socioeconomic status (SES), marital status, the child’s birth weight, and information on breastfeeding were extracted from the parent’s questionnaires. SES was based on net household income, and was dichotomized into “low SES” (<2000 euros/month) and “middle/high SES” (≥2000 euros/month) using the mean monthly general labor income of 2014 as the cut-off point.^32^ Ethnicity (“both parents born in the Netherlands” and “at least one parent not born in the Netherlands”), marital status (“parents are together” and “parents separated”), and breastfeeding (“breastfed” and “not breastfed”) were also dichotomized. Parental education was categorized into three classes: “up to lower level secondary education,” “higher level secondary education,” and “at least a bachelor diploma.”

In order to analyze the frequency and type of consultations, respiratory consultations in general were defined as all ICPC codes with the letter “R.” Asthma-like consultations were defined as ICPC codes R02 (shortness of breath), R03 (wheezing), and R96 (asthma). Respiratory allergy-related consultations were defined as ICPC code R02, R03, R96, and R97 (allergic rhinitis). Respiratory inflammatory consultations were defined as ICPC codes R05 (cough), and R71 up to R83 (respiratory inflammatory codes).

### Outcome measures

The primary outcome measures in this study were the frequency and type of (specific) respiratory consultations during the 2-year follow-up in underweight, normal-weight, and overweight children. Secondary outcome measure was the overall number of consultations.

### Sample size calculation

The incidence of self-reported respiratory diseases in children with overweight is 0.311, and in children of normal-weight the incidence is 0.217.^33^ When using the formula of Fleiss with a two-sided significance level of 0.05 and a power of 90%, the sample size is 461 children in each group.^28,34^ Taking about 10% of drop-outs into account the number of participants in each group is 500.

### Statistical analysis

The independent *T* test was used to test for differences in baseline characteristics between the included and excluded children. The analysis of variance test was used to compare baseline characteristics between the three different weight status groups. Analysis for collinearity between potential confounders showed no collinearity between confounders; therefore, all analyses were adjusted for gender, age, ethnicity, SES, and breastfeeding. Missing data on confounders (8.5%) were handled using multiple imputation with 10 iterations. Logistic regression analyses were used to assess the association between weight status and the presence of respiratory consultations, asthma-like consultations, respiratory inflammatory consultations, and respiratory allergy-related consultations during the 2-year follow-up. Negative binomial regression was used to test the association between weight status and the frequency of respiratory consultations, asthma-like consultations, respiratory inflammatory consultations, and respiratory allergy-related consultations during the 2-year follow-up. Subgroup analyses were performed in three different age categories: 2–6, 6–12, and 12–18 years old. Sensitivity analyses were performed to test for differences in frequencies of respiratory consultations between normal-weight and underweight children, using a stricter cut-off for underweight status so that the 7.4% underweight prevalence in the Netherlands was simulated.^14^

*P* values <0.05 were considered statistically significant. Adjusted ORs with a 95% CI were used to determine the strength of associations. Data were analyzed using IBM SPSS Statistics 21/24.

## Supplementary information

Supplementary Table 1

## Data Availability

The datasets generated during and/or analyzed during the current study are available from the corresponding author on reasonable request.

## Supplementary information

**Supplementary Information** accompanies the paper on the *npj Primary Care Respiratory Medicine* website (10.1038/s41533-019-0131-0).
