# Supplementary material for: Differences in respiratory consultations in primary care between underweight, normal-weight, and overweight children
Source: NPJ Prim Care Respir Med. 2019 May 3;29:15. doi: 10.1038/s41533-019-0131-0 (PMC6499776; doi:10.1038/s41533-019-0131-0)
Supplement: Supplementary file 1 — Supplementary Table 1 [file 41533_2019_131_MOESM1_ESM.pdf]

Supplementary Table 1 – ICPC R-codes

ICPC-code	Diagnosis
**R01**	Pain respiratory system
**R02**	Shortness of breath/dyspnea
**R03**	Wheezing
**R04**	Breathing problem, other
**R04.01**	Snoring
**R05**	Cough
**R06**	Nose bleed/epistaxis
**R07**	Sneezing/nasal congestion/runny nose
**R08**	Nose symptom/complaint other
**R09**	Sinus symptom/complaint (including pain)
**R21**	Throat symptom/complaint
**R21.01**	Sore throat
**R22**	Tonsils symptom/complaint
**R23**	Voice symptom/complaint
**R24**	Hemoptysis
**R25**	Sputum/phlegm abnormal
**R26**	Fear of cancer respiratory system
**R27**	Fear of respiratory disease, other
**R28**	Limited function/disability respiratory
**R29**	Respiratory symptom/complaint other
**R29.01**	Hiccup
**R70**	Tuberculosis respiratory
**R71**	Whooping cough
**R72**	Strep throat/scarlet fever
**R72.01**	Strep throat
**R72.02**	Scarlet fever
**R73**	Boil/abscess nose
**R74**	Upper respiratory infection acute
**R74.01**	Common cold
**R74.02**	Acute pharyngitis
**R75**	Sinusitis acute/chronic
**R75.01**	Sinusitis acute
**R75.02**	Sinusitis chronic
**R76**	Tonsillitis acute/peritonsillar abscess
**R76.01**	Tonsillitis acute
**R76.02**	Peritonsillar abscess
**R77**	Laryngitis/tracheitis acute
**R77.01**	Laryngitis subglottic/pseudocroup
**R77.02**	Epiglottitis acute
**R78**	Acute bronchitis/bronchiolitis
**R80**	Influenza
**R81**	Pneumonia
**R81.01**	Legionella pneumonia
**R82**	Pleurisy
**R83**	Respiratory infection other

**R83.01**	Diphtheria
**R83.02**	Sarcoidosis
**R84**	Malignant neoplasm bronchus/lung
**R85**	Malignant neoplasm respiratory, other
**R86**	Benign neoplasm respiratory
**R87**	Foreign body nose/larynx/bronchia
**R88**	Injury respiratory other
**R89**	Congenital anomaly respiratory
**R90**	Hypertrophy/chronic infection tonsils/adenoids
**R91**	Bronchitis chronic/bronchiectasis
**R91.01**	Bronchitis chronic
**R91.02**	Bronchiectasis
**R93**	Pleural effusion
**R95**	Emphysema/COPD
**R96**	Asthma
**R96.01**	Hyper reactivity respiratory
**R96.02**	Allergy asthma
**R97**	Hay fever/allergic rhinitis
**R98**	Hyperventilation
**R99**	Respiratory disease other
**R99.01**	Deviation septum nose
**R99.02**	Nasal polyp
**R99.03**	Vocal cord polyp/vocal cord lump
**R99.04**	Pneumothorax
**R99.05**	Aspiration pneumonia
**R99.06**	Pneumoconiosis
